# Association of Genetic Variants of KCNJ11 and KCNQ1 Genes with Risk of Type 2 Diabetes Mellitus (T2DM) in the Indian Population: A Case-Control Study

**DOI:** 10.1155/2020/5924756

**Published:** 2020-10-10

**Authors:** Vasiuddin Khan, Amit Kumar Verma, Deepti Bhatt, Shahbaz Khan, Rameez Hasan, Yamini Goyal, Sowmya Ramachandran, Mohammed A. Alsahli, Arshad Husain Rahmani, Ahmad Almatroudi, M. Y. Shareef, Babita Meena, Kapil Dev

**Affiliations:** ^1^Department of Biotechnology, Jamia Millia Islamia, New Delhi, India; ^2^Department of Medical Laboratories, College of Applied Medical Sciences, Qassim University, Buraidah, Saudi Arabia; ^3^Faculty of Dentistry, Jamia Millia Islamia, New Delhi, India

## Abstract

Type 2 diabetes mellitus (T2DM) is a polygenic metabolic disease described by hyperglycemia, which is caused by insulin resistance or reduced insulin secretion. The interaction between various genetic variants and environmental factors triggers T2DM. The aim of this study was to find risk associated with genetic variants rs5210 and rs2237895 of KCNJ11 and KCNQ1 genes, respectively, in the development of T2DM in the Indian population. A total number of 300 cases of T2DM and 100 control samples were studied to find the polymorphism in KCNJ11 and KCNQ1 through PCR-RFLP. The genotype and allele frequencies in T2DM cases were significantly different compared to the control population. KCNJ11 rs5210 and KCNQ1 rs2237895 variants were found to be significantly associated with risk of T2DM in dominant (KCNJ11: OR, 2.07; 95% CI, 1.30–3.27; *p* − 0.001; KCNQ1: OR, 2.33; 95% CI, 1.46–3.70; *p* − 0.0003) and codominant models (KCNJ11: OR, 1.76; 95% CI, 1.09–2.84; *p* − 0.020; KCNQ1: OR, 1.85; 95% CI, 1.16–2.95; *p* − 0.009). We also compared clinicopathological characteristics between cases and control and observed a significant difference in all the parameters except HDL, gender, and family history. In this study, clinicopathological data with a carrier of a variant allele of both KCNJ11 and KCNQ1 genes were also analysed, and a significant association was found between the carrier of a variant allele with gender and PPG in KCNJ11 and with triglyceride in KCNQ1. We confirm the significant association of KCNJ11 (rs5210) and KCNQ1 (rs2237895) gene polymorphism with T2DM, indicating the role of these variants in developing risk for T2DM in Indian population.

## 1. Introduction

Type 2 Diabetes is a chronic metabolic disorder described by reduced insulin secretion and insulin action and hypergycemia. Among different types of diabetes, T2DM is the most common wherein multiple environmental and genetic factors are associated with the development of this disease [1, 2]. As per the International Diabetes Federation's (IDF) Diabetes Atlas, 8^th^ edition, it has been estimated that the number of diabetes patients is expected to increase from 425 million in 2017 to 629 million by 2045. India is home to 74 million people with diabetes mellitus, with approximately 8.7% prevalence among the adult population, and considered as the “diabetes capital” of the world (IDF, 8^th^ edition). Genome-wide association studies (GWAS) found various susceptibility genes for diabetes [3–5]. However, the mechanisms these genes follow for the development of diabetes are still not clear. Many gene polymorphism findings demonstrated the association of various SNPs in the pathogenesis of T2DM, and in different populations, each SNP may exhibit dissimilar association with T2DM. Therefore, there is a necessity to discover various genetic risk markers of T2DM for its prevention and treatment.

KCNJ11 and KCNQ1 genes have gained significant attention as a potential candidate for T2DM susceptibility because of their position and function in regulating glucose-stimulated insulin secretion. The KCNJ11 gene is located at 11p15.1 on the human chromosome and lacks intronic sequences; this gene is a member of the potassium channel gene family [6]. The KCNJ11 gene encodes Kir6.2, an inward-rectifier potassium ion channel, which forms a KATP channel with sulfonylurea receptor 1 (SUR1). This KATP channel, through glucose metabolism, controls insulin secretion and production [7]. The KCNQ1 gene is located at the 11th chromosome 11p15.5, approximately 404 kb, and consists of 17 exons [8]. The potassium voltage-gated channel KQT-like subfamily, member 1 (KCNQ1), encodes the pore-forming subunit of KvLQT1, voltage-gated potassium channel, and inhibition of this channel by KCNQ1 inhibitors 293B increased insulin secretion [9], indicating the important role of the KCNQ1 channel in regulating insulin secretion. KCNJ11 and KCNQ1 are ATP-sensitive K^+^ channels and play crucial role in regulating insulin-secreting *β* cells making them potential susceptibility genes for T2DM. Therefore, the present case-control study was conducted to assess the association of KCNJ11 (rs5210) and KCNQ1 (rs2237895) gene variants with T2DM risk in Indian population and its association with various clinicopathological characteristics to understand the implications of ethnic diversity on the onset of T2DM.

## 2. Materials and Methods

### 2.1. Ethical Approval and Study Subjects

Collection of clinical data in pretext Performa and sample collection of the diabetic patients and control/nondiabetic of North Indian origin from Ansari Health Center Jamia Millia Islamia New Delhi were performed with due consent, after getting Ethical clearance from the Institute Ethical Committee, Jamia Millia Islamia, New Delhi (Proposal No. 17/9/14/JMI/IEC/2015 dated 14/01/2016). The present cohort study was conducted in the Department of Biotechnology, Jamia Millia Islamia, New Delhi, and a total of 400 samples (300 cases and 100 controls) were collected, fulfilling all the relevant selection criteria.

All the study participants had undergone detailed clinical investigation, and written informed consent was collected from the patients before their participation. Various clinicopathological characteristics such as age, BMI, PPG, FPG, FPI, HbA1c, T-cholesterol, systolic BP, diastolic BP, triglycerides, LDL-C,HDL-C, gender, family history, smoking, and alcohol consumption were included in this study.

### 2.2. Single Nucleotide Polymorphism (SNP) Genotyping by PCR-RFLP

Genomic DNA was extracted from a fresh blood sample using the phenol chloroform method. The primers used for amplification were as follows: KCNJ11 (Forward: 5′- ATCCAGGGTGTTACAAGGCA-3′; reverse: 5′-TTTCAGGGACCAAGTAGAGCTG-3′) and KCNQ1 (Forward 5′-ATCCAGGGTGTTACAAGGCA-3'; reverse 5'- TTTCAGGGACCAAGTAGAGCT -3') in 20 ul of the reaction mixture volume using the Go TAQ Green Master Mix (Promega). For KCNJ11 rs5210, PCR conditions were as follows: an initial denaturation of 95°C for 5 minutes, followed by 35 cycles of 95°C for 30 sec, 60°C for 30 sec, and 72°C for 30 sec, and final extension of 72°C for 5 min, and for KCNQ1 rs2237895, an initial denaturation of 94°C for 5 minutes, 35 cycles (94°C for 30 s, 58°C for 30 s, and 72°C for 30 s), and final extension of 72°C for 10 m in a PeqSTAR 96 universal gradient thermocycler (Peqlab,VWR). Amplified products were, then, electrophoresed on 2% agarose gel, and images were captured by using the gel documentation system (Biorad).

**Figure 1:**
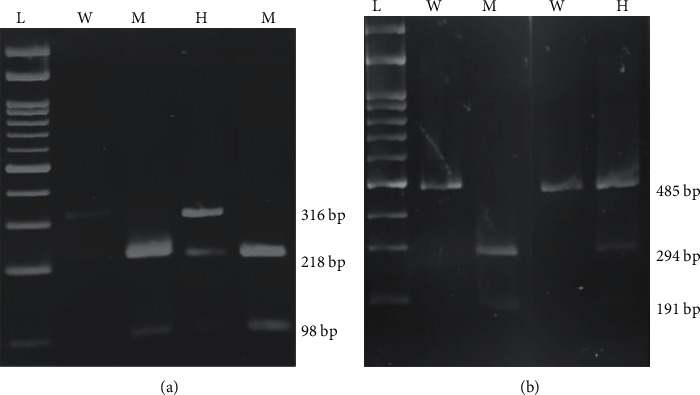
3% agarose gel electrophoresis for the digested PCR product with Hpy188III (NEB) and Ava I (NEB), respectively. (a) KCNJ11, L-100 bp DNA ladder; W- Homo Wild (A/A); M- Homo Mutant (G/G); H- Hetero (A/G) and (b) KCNQ1, L-100 bp DNA ladder; W- Homo Wild (A/A); M- Homo Mutant (C/C); H- Hetero (A/C).

KCNJ11 (6956A > G) and KCNQ1 (395974 A > C) polymorphism were detected by using a 6 ul PCR product with 2.5 units of restriction enzymes Hpy188III (NEB) and Ava I (NEB), respectively, incubated overnight at 37°C. Hpy188III digested the PCR product of the KCNJ11 gene; wild type allele (A/A) yielded 1 band of 316 bp; heterozygous (A/G) yielded 3 bands of 316 bp, 218 bp, and 98 bp, and risk allele (G/G) yielded two bands of 218 bp and 98 bp (Figure 1(a)), while Ava I digested the PCR product of the KCNQ1 gene; wild type allele (A/A) produced one band of 485 bp; heterozygous (A/C) produced three bands of 485 bp, 294 bp, and 191 bp, and risk allele (C/C) produced two bands of 294 bp and 191 bp (Figure 1(b)). The digested PCR products were subjected to electrophoresis in a 3% agarose gel, as shown in Figures 1(a) and 1(b)

### 2.3. Statistical Analysis

Statistical analysis was performed by SPSS Software (21.0 Version, IBM, United States). The chi-square test was applied for comparing genotype and allele frequencies for statistical significance between diabetic patients and controls. Data were presented as mean ± SD or as the number of cases. Comparative analysis of clinicopathological characteristics between cases and control was performed by Student's *t*-test, while the chi-square test was used for gender, family history, smoking, and alcohol consumption. The level of significance was set at 95% (i.e., *p*  <  0.05).

## 3. Results

### 3.1. Clinicopathological Characteristics in T2DM Cases and Control

**Figure 2:**
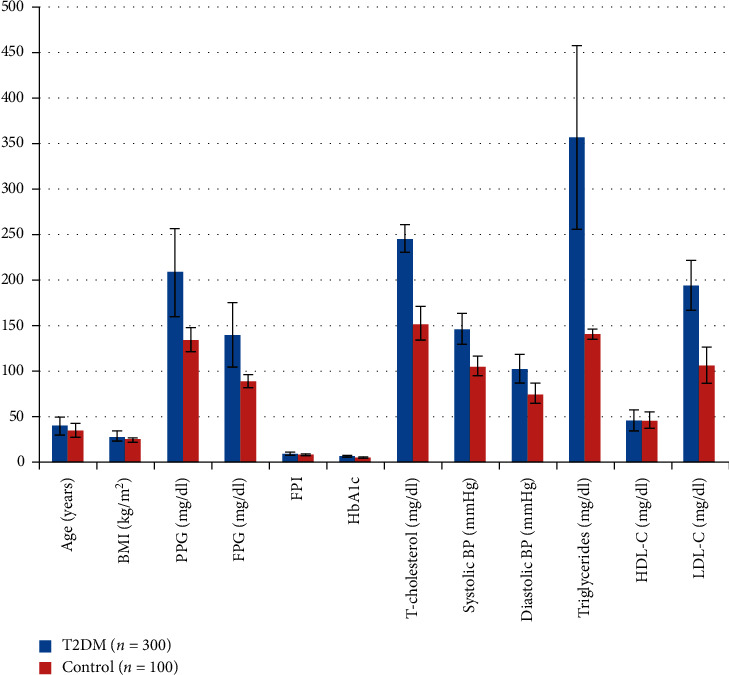
Graphical representation of clinicopathological characteristics of T2DM patients and controls.

Various clinicopathological characteristics were compared between cases and control by using Student's *t*-test and the chi-square test. The average age of T2DM cases was (40.33 ± 9.76) years, whereas it was (35.29 ± 7.96) years in controls. Compared to control, T2DM cases had significantly higher BMI, PPG, FPG, FPI, HbA1c, T-cholesterol, systolic BP, diastolic BP, triglycerides, and LDL-C. A significant difference was found in the smoking and alcohol consumption between cases and control, while no significant difference was found in HDL, gender, and family history, as depicted in Table 1 and Figure 2.

### 3.2. Genotype Distribution and Allelic Frequencies of KCNJ11 and KCNQ1 Genes among T2DM Cases and Controls

The genotype and allele distribution of the KCNJ11 gene is shown in Table 2. The distribution of the genotypes was significantly different between T2DM cases and controls. When the AA genotype was set as the reference, both AG and GG genotypes were found to be associated with increased risk for T2DM (OR, 2.05; 95% CI, 1.25–3.37; *p* − 0.004 for AG; and OR, 2.12; 95% CI, 0.99–4.5; *p* − 0.046 for GG, respectively, Table 2). The statistical analysis of the observed genotypic frequencies for KCNJ11 (rs5210) showed a significant association (*p* − 0.007). The frequency of KCNJ11 rs5210 G allele was significantly higher in cases than in controls (37% vs. 26%, respectively). When the A allele of rs5210 was set as reference, the G allele of KCNJ11 contributed to increased risk of T2DM (OR, 1.67; 95% CI, 1.16–2.38; *p* − 0.004). We found a significant relationship between KCNJ11 rs5210 A** ⟶ **G gene polymorphism and T2DM risk under the dominant (OR, 2.07; 95% CI, 1.30–3.27; *p* − 0.001) and codominant models (OR, 1.76; 95% CI, 1.09–2.84; *p* − 0.020), whereas no significant relationship was found under the recessive model (OR, 1.54; 95% CI, 0.74–3.20; *p* − 0.239).

The genotype and allele distribution of the KCNQ1 gene are shown in Table 3. The distribution of the genotypes was significantly different between T2DM cases and controls. When the AA genotype was set as the reference, both AC and CC genotypes were found to be associated with increased risk for T2DM (OR, 2.36; 95% CI, 1.43–3.89; *p* − 0.0007 for AC; and OR, 2.26; 95% CI, 1.14–4.46; *p* − 0.017 for CC). The statistical analysis of the observed genotypic frequencies for KCNQ1 showed a significant association (*p* − 0.001). The frequency of KCNQ1 rs2237895C allele was significantly higher in cases than in controls (44% vs. 32%, respectively). When the A allele of rs2237895 was set as reference, the C allele of KCNQ1 contributed to the increased risk of T2DM (OR, 1.66; 95% CI, 1.19–2.34; *p* − 0.003). We found a significant relationship between KCNQ1 rs2237895 A⟶C gene polymorphism and T2DM risk under the dominant model (OR, 2.33; 95% CI, 1.46–3.70; *p* − 0.0003) and codominant model (OR, 1.85; 95% CI, 1.16–2.95; *p* − 0.009), whereas no significant relationship was found under the recessive model (OR, 1.44; 95% CI, 0.76–2.71; *p* − 0.259).

### 3.3. Observed and Expected Genotypes of KCNJ11 and KCNQ1 Polymorphism in Control and Cases

The observed and expected genotype frequencies of KCNJ11 and KCNQ1 gene polymorphism in controls and cases showed no deviation from the Hardy–Weinberg equilibrium. The chi-square test demonstrated that there was no significant deviation from the Hardy–Weinberg equilibrium for KCNJ11 and KCNQ1 SNP genotypes (*p* > 0.05), as shown in Table 4.

### 3.4. Association of the Clinicopathological Characteristic of Type 2 Diabetes Patients with the Carrier of a Variant Allele of KCNJ11 and KCNQ1 Genes

**Figure 3:**
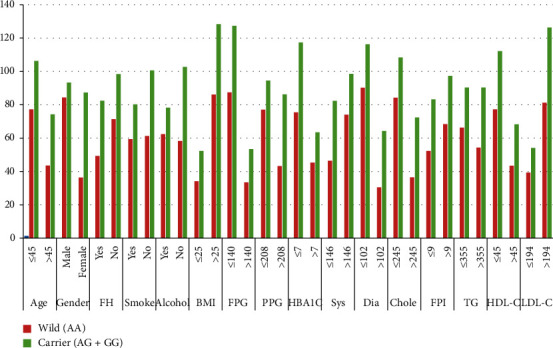
Graphical representation of clinicopathological characteristics of T2DM patients between wild and carrier of a variant allele of the KCNJ11 gene.

We analysed the clinicopathological data with the carrier of a variant allele (AG + GG) of the KCNJ11 gene and found out that there was no statistically significant association between the carrier of a variant allele with age, family history, smoking, alcohol consumption, BMI, FPG, HB1Ac, systolic BP, diastolic BP, cholesterol, FPI, triglyceride, HDL, and LDL. We observed a significant association of gender and PPG with the carrier of a variant allele (AG + GG), as shown in Table 5 and Figure 3 (the result is significant at *p* > 0.05).

**Figure 4:**
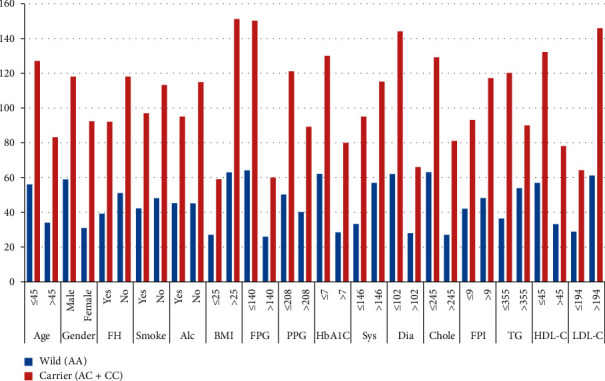
Graphical representation of clinicopathological characteristics of T2DM patients between wild and carrier of a variant allele of the KCNQ1 gene.

Similarly, clinicopathological data with the carrier of a variant allele (AC + CC) of the KCNQ1 gene were analysed, and we found out that there was no statistically significant association between the carrier of a variant allele with age, gender, family history, smoking, alcohol consumption, BMI, FPG, PPG, HB1Ac, systolic BP, diastolic BP, cholesterol, FPI, HDL-C, and LDL-C. We found a significant association of triglyceride with the carrier of a variant allele (AC + CC), as shown in Table 6 and Figure 4 (the result is significant at *p* > 0.05).

### 3.5. Combined Effect of KCNJ11 + KCNQ1 Genotypes on Clinicopathological Parameters

The effect of KCNJ11 + KCNQ1 genotypes together had a significant impact on the regulation of PPG, FPG, cholesterol, LDL, and diastolic BP (Table 7).

### 3.6. Regression Analysis: Dependent Variable Cases and Control

Regression analysis was performed to check the involvement of other variables with the occurrence of T2DM. It was observed that the variables such as age, BMI, PPG, FPG,FPI, HbA1C, cholesterol, LDL, systolic BP, diastolic BP, and triglycerides showed a significant association with the study subject and *R*^2^ showed an association with T2DM (Table 8), while no association was observed with gender and HDL.

## 4. Discussion

In the present case-control study, we investigated the association of SNPs (rs5210) and (rs2237895) within the KCNJ11 and KCNQ1 genes, respectively, with the susceptibility to T2DM in Indian population. The KCNJ11 polymorphism association with T2DM risk has been extensively studied among European population; however, their relationship in the Indian subcontinent is yet to be validated. Various GWAS also demonstrated that several KCNQ1 variants (i.e., rs2237892, rs2237895, and rs2237897) are associated with T2DM risk and reduced insulin secretion in Europeans, Asians, and American Indians [10–12]. However, studies in different races and regions have demonstrated different findings. It has also been stated that Indians might have different genetic predisposition to diabetes than Europeans [13]. In this study, we compared various clinicopathological characteristics between cases and control and observed a significant difference in all the parameters except HDL, gender, and family history. Dorman and Bunker. suggested that people with a T2DM family history are three times more susceptible to this disease [14]. However, in our study, we have not observed any association of family history with T2DM risk between cases and control. However, family history is considered as a strong and independent risk factor for diabetes [15–19].

In humans, the significance of KCNJ11 in insulin secretion was suggested by its function in permanent neonatal diabetes [20] and familial persistent hyperinsulinemic hypoglycaemia of infancy [21]. KCNJ11 is considered as a promising candidate susceptibility gene for T2DM due to the protein encoded by KCNJ11, which is crucial for pancreatic beta-cell function. In the present study, we observed a significant difference in the distribution of KCNJ11 genotypes among T2DM cases and healthy controls and higher risk allele distribution was observed among cases as compared to healthy controls. Also, the KCNJ11 rs5210 variant demonstrated a significant association between T2DM cases and controls under dominant and codominant models. Our findings indicated an association of the KCNJ11 rs5210 polymorphism with T2DM in Indian patients. Our results were found to be similar to other studies which confirmed the association of KCNJ11 variant 3p + 215 (rs5210) with T2DM [22, 23]. Similarly, meta-analysis in the East Asian population and genotypic and allelic contrast also suggested a significant association of KCNJ11 and T2DM for rs5210 [24]. Furthermore, the rs5210 variant of the KCNJ11 gene was indicated to be related with T2DM risk in meta-analysis of 5 studies, and it was significantly heterogeneous (*p*=0.02) [25]. Similar results were also observed among the Mexican, Finnish, and Korean populations [26–28]. Our results were found to be inconsistent with other studies in which no association was found between KCNJ11 E23K polymorphism and T2DM in Iranian [29], Czech [30], Moroccan [31], and Mongolian population [32].

KCNQ1 comprises of 676 amino acids with 1 ion-selective P loop and 6 transmembrane regions. The P loop is composed of 4 identical *α* subunits, which is an ion-filter duct. It consists of a porous structure which is highly conservative leading to higher selectivity to potassium. The KCNQ1 knockout mouse showed prominent increase in insulin sensitivity, suggesting that KCNQ1 may act as a novel element affecting insulin sensitivity through glucose metabolism [33]. In our study, the genotype and allele distribution of KCNQ1 rs2237895 showed a significant difference between T2DM cases and controls and higher risk allele distribution was observed among cases as compared to healthy controls. Also, the KCNQ1 rs2237895 variant showed a significant association between T2DM cases and controls under dominant and codominant models. Our findings indicated an association of the KCNQ1 rs2237895 polymorphism with T2DM in Indian patients. Our findings were found to be consistent with the previous study in which KCNQ1 gene polymorphism (rs2074196, rs2237892, rs2237895, rs2283228, and rs2237897) was linked with T2DM risk among Japanese populations in 2 independent genome-wide association studies [3, 10]. Furthermore, in European population, according to GWA scans and replication studies, SNPs (rs151290, rs2237892, and rs2237895) of the KCNQ1 were indicated to be a genetic risk factor for T2DM [3, 10, 34–36]. In addition, many previous studies proposed that various variants of KCNQ1 rs2237895, rs2237892, rs2237897, and rs151290 showed an association with T2DM in China [37], Germany [35], Pakistan, and the Netherlands [38, 39].

In East Asian origin patients, the KCNQ1 gene has been found to play a significant role in contributing to T2D susceptibility [3, 10, 40]. Some studies were contradictory to our finding which did not find any association of KCNQ1 variants (rs2237892 and rs2273895) and T2DM risk [41, 42]. A study on the South Indian population from Hyderabad also showed a significant association of KCNJ11 (rs5210) and KCNQ1 (rs2283228) with T2DM risk, which corroborates with our results [43]. However, we did not come across any such study investigating the association of KCNJ11 (rs5210) and KCNQ1 (rs2237895) gene polymorphism with T2DM risk in North Indian origin patients.

Additionally, we analysed clinicopathological data with the carrier of a variant allele of KCNJ11 and KCNQ1. We found an association between the carrier of a variant allele with gender and PPG in KCNJ11 and with triglyceride in KCNQ1.Chen et al. also suggested that KCNQ1 was associated with triglyceride levels in the Chinese Han population [44].This study will help in understanding the genetic background of T2DM, which is very essential for the identification of high-risk individuals and to include prevention plan in these high-risk individuals and general population.

## 5. Conclusions

In the present study, we identify a significant association of KCNJ11 (rs5210) and KCNQ11 (rs2237895) gene polymorphism with T2DM risk, suggesting the role of these variants in the increased risk of developing T2DM in the Indian population. We also found that the G allele of rs5210 and C allele of rs2237895 polymorphism of KCNJ11 and KCNQ1, respectively, are the major risk factors that confer susceptibility to T2DM. Furthermore, these genetic variants will enable us to find the risk prediction of T2DM development and its underline pathogenesis, which will be applicable for individualized treatment of T2DM.

## Figures and Tables

**Table 1:** Clinicopathological characteristics of T2DM patients and controls.

Characteristic	T2DM (*n* = 300)	Control (*n* = 100)	*p* value
Age (years)	40.33 ± 9.76	35.29 ± 7.96	<0.0001^*∗*^
BMI (kg/m^2^)	28.8 ± 5.22	24.83 ± 2.33	<0.0001^*∗*^
PPG (mg/dl)	208.5 ± 48.49	135 ± 13.02	<0.0001^*∗*^
FPG (mg/dl)	140 ± 35.64	90.22 ± 7.11	<0.0001^*∗*^
FPI	9.6 ± 1.35	8.66 ± 0.71	<0.0001^*∗*^
HbA1c	7.12 ± 1.03	5.75 ± 0.54	<0.0001^*∗*^
T-cholesterol (mg/dl)	245.58 ± 15.14	152.63 ± 18.82	<0.0001^*∗*^
Systolic BP (mmHg)	146.79 ± 17.05	106.07 ± 10.39	<0.0001^*∗*^
Diastolic BP (mmHg)	102.87 ± 16.19	75.85 ± 10.91	<0.0001^*∗*^
Triglycerides(mg/dl)	356.32 ± 100.48	140.98 ± 5.52	<0.0001^*∗*^
HDL-C (mg/dl)	45.89 ± 11.47	46.21 ± 8.7	0.7985
LDL-C (mg/dl)	194.60 ± 27.38	106.42 ± 19.92	<0.0001^*∗*^
Gender (male/female)	177/123	68/32	0.11
Family history (yes/no)	131/169	39/61	0.414
Smoking (yes/no)	139/161	15/85	<0.0001^*∗*^
Alcohol consumption (yes/no)	140/160	10/90	<0.0001^*∗*^

Data are presented as mean ± SD except gender, family history, smoking, and alcohol consumption. *p* values were calculated by Student's *t*-test, while the Chi-square test was used for gender, family history, smoking, and alcohol consumption. ^*∗*^Significant at *p* < 0.05. BMI: body mass index; FPG: fasting plasma glucose; FPI: fasting plasma insulin; HbA1c: haemoglobin A_1c_ HDL: high-density lipoprotein; LDL: low-density lipoprotein.

**Table 2:** Genotypic and allelic distribution and association analysis of KCNJ11 rs5210 gene polymorphism and risk of T2DM under different genetic models.

Genotype/allele		Cases (*n* = 300)	Control (*n* = 100)	Odds ratio (95% CI)	*p* value
AA		120 (40%)	58 (58%)	Ref	Ref
AG		136 (45.3%)	32 (32%)	2.05 (1.25–3.37)	0.004^*∗*^
GG		44 (14.6%)	10 (10%)	2.12 (0.99–4.5)	0.046^*∗*^
*p* value −0.007^*∗*^					
Recessive model	GG	44	10	1.54 (0.74–3.20)	0.239
	AG + AA	256	90		
Dominant model	AG + GG	180	42	2.07 (1.30–3.27)	0.001^*∗*^
	AA	120	58		
Codominant model	AG	136	32	1.76 (1.09–2.84)	0.020^*∗*^
	AA + GG	164	68		
Allele					
A		378 (63%)	148 (74%)	1.67(1.16–2.38)	0.004^*∗*^
G		222 (37%)	52 (26%)		

OR: odds ratio, CI: confidence interval, *n*: number in sample.^*∗*^Significant at *p* < 0.05.

**Table 3:** Genotypic and allelic distribution and association analysis of KCNQ1 rs2237895 genes polymorphism and risk of T2DM under different genetic models.

Genotype		Cases (*n* = 300)	Control (*n* = 100)	Odds ratio (95% CI)	*p* value
AA		90 (30%)	50 (50%)	Ref	Ref
AC		153 (51%)	36 (36%)	2.36 (1.43–3.89)	0.0007^*∗*^
CC		57 (19%)	14 (14%)	2.26 (1.14–4.46)	0.017^*∗*^
*p* value −0.001^*∗*^					
Recessive model	CC	57	14	1.44 (0.76–2.71)	0.259
	AC + AA	243	86		
Dominant model	AC + CC	210	50	2.33 (1.46–3.70)	0.0003^*∗*^
	AA	90	50		
Codominant model	AC	153	36	1.85 (1.16–2.95)	0.009^*∗*^
	AA + CC	147	64		
Allele					
A		336 (56%)	136 (68%)	1.66 (1.19 to 2.34)	0.003^*∗*^
C		264 (44%)	64 (32%)		

**Table 4:** Observed and expected genotypes of KCNJ11 and KCNQ1 polymorphism in control and cases.

Genotype	Observed genotype	Expected genotype	*p* value	Chi square (*X*^2^)
Control				

KCNJ11 rs5210				

AA	58	54.76	0.2422	2.836
AG	32	38.48		
GG	10	6.76		

KCNQ1 rs2237895				

AA	50	46.24	0.2247	2.986
AC	36	43.52		
CC	14	10.24		

Cases				

KCNJ11 rs5210				
AA	120	117.8133	0.8645	0.2912
AG	136	140.3733		
GG	44	41.8133		
KCNQ1 rs2237895				
AA	90	92.4075	0.8535	0.3167
AC	153	148.185		
CC	57	59.4075		

**Table 5:** Clinicopathological characteristics of T2DM patients between wild and carrier of a variant allele of the KCNJ11 gene.

Characteristic	Total	Wild (AA)	Carrier (AG + GG)	*p* value
Age				
≤45	183	77	106	0.358
>45	117	43	74	

Gender				
Male	177	84	93	0.0015^*∗*^
Female	123	36	87	

Family history				
Yes	131	49	82	0.419
No	169	71	98	

Smoking				
Yes	139	59	80	0.421
No	161	68	100	

Alcohol consumption				
Yes	140	62	78	0.156
No	160	5	102	

BMI				
≤25	86	34	52	0.916
>25	214	83	128	

FPG				
≤140	214	87	127	0.715
>140	86	3	53	

PPG				
≤208	171	77	94	0.040^*∗*^
>208	129	43	86	

HBA1C				
≤7	192	75	117	0.658
>7	108	45	63	

Systolic BP				
≤146	128	46	82	0.215
>146	172	74	98	

Diastolic BP				
≤102	206	90	116	0.053
>102	94	30	64	

Cholesterol				
≤245	192	84	108	0.077
>245	108	36	72	

FPI				
≤9	135	52	83	0.635
>9	165	68	97	

Triglyceride				
≤355	156	66	90	0.395
>355	144	54	90	

HDL-C				
≤45	189	77	112	0.732
>45	111	43	68	

LDL-C				
≤194	93	39	54	0.646
>194	207	81	126	

*p* values were determined by the chi-square test. ^*∗*^Significant association at *p* > 0.05.

**Table 6:** Clinicopathological characteristics of T2DM patients between wild and carrier of a variant allele of the KCNQ1 gene.

Characteristic	Total	Wild (AA)	Carrier (AC + CC)	*p* value
Age				
≤45	183	56	127	0.77
>45	117	34	83	

Gender				
Male	177	59	118	0.130
Female	123	31	92	

Family history				
Yes	131	39	92	0.939
No	169	51	118	

Smoking				
Yes	139	42	97	0.939
No	161	48	113	

Alcohol consumption				
Yes	140	45	95	0.448
No	160	45	115	

BMI				
≤25	86	27	59	0.738
>25	214	63	151	

FPG				
≤140	214	64	150	0.955
>140	86	26	60	

PPG				
≤208	171	50	121	0.740
>208	129	40	89	

HBA1C				
≤7	192	62	130	0.248
>7	108	28	80	

Systolic BP				
≤146	128	33	95	0.168
>146	172	57	115	

Diastolic BP				
≤102	206	62	144	0.956
>102	94	28	66	

Cholesterol				
≤245	192	63	129	0.156
>245	108	27	81	

FPI				
≤9	135	42	93	0.704
>9	165	48	117	

Triglyceride				
≤355	156	36	120	0.006^*∗*^
>355	144	54	90	

HDL-C				
≤45	189	57	132	0.937
>45	111	33	78	

LDL-C				
≤194	93	29	64	0.764
>194	207	61	146	

*p* values were determined by the chi-square test. ^*∗*^Significant association at *p* > 0.05.

**Table 7:** Combined effect of KCNJ11 + KCNQ1 genotypes on clinicopathological parameters.

Parameters	Genotype variables KCNJ11 + KCNQ1	Mean ± SD	*p* value

BMI	(AA + AA)	27.79 ± 4.47	0.069
	(AG + AC)	29.24 ± 5.07	
	(GG + CC)	25.99 ± 3.37	
	(AG + CC)	27.88 ± 5.49	
	(AG + AA)	27.45 ± 4.8	
	(GG + AA)	27.47 ± 5.00	

PPG	(AA + AA)	177.41 ± 53.14	0.003^*∗*^
	(AG + AC)	193.88 ± 39.85	
	(GG + CC)	177.59 ± 47.63	
	(AG + CC)	210.09 ± 68.61	
	(AG + AA)	184.07 ± 46.72	
	(GG + AA)	196.50 ± 62.67	

FPG	(AA + AA)	121.12 ± 40.06	0.030^*∗*^
	(AG + AC)	132.55 ± 38.45	
	(GG + CC)	120.45 ± 26.15	
	(AG + CC)	132.07 ± 43.79	
	(AG + AA)	122.18 ± 30.65	
	(GG + AA)	134.38 ± 42.72	

FPI	(AA + AA)	9.04 ± 1.20	0.29
	(AG + AC)	9.54 ± 1.41	
	(GG + CC)	8.98 ± 1.14	
	(AG + CC)	9.42 ± 1.24	
	(AG + AA)	9.39 ± 1.23	
	(GG + AA)	9.42 ± 1.40	

HbA1C	(AA + AA)	6.57 ± 1.10	0.238
	(AG + AC)	6.94 ± 1.12	
	(GG + CC)	6.59 ± 0.93	
	(AG + CC)	6.79 ± 1.08	
	(AG + AA)	6.74 ± 1.08	
	(GG + AA)	6.92 ± 1.24	

Cholesterol	(AA + AA)	211.97 ± 46.99	0.009^*∗*^
	(AG + AC)	230.80 ± 38.24	
	(GG + CC)	218.63 ± 52.46	
	(AG + CC)	234.34 ± 36.31	
	(AG + AA)	216.69 ± 44.70	
	(GG + AA)	223.84 ± 45.10	

HDL	(AA + AA)	46.69 ± 9.18	0.117
	(AG + AC)	45.03 ± 10.48	
	(GG + CC)	47.72 ± 9.52	
	(AG + CC)	45.39 ± 12.97	
	(AG + AA)	46.80 ± 10.20	
	(GG + AA)	44.23 ± 12.98	

LDL	(AA + AA)	159.88 ± 48.49	0.005^*∗*^
	(AG + AC)	182.14 ± 41.31	
	(GG + CC)	170.90 ± 53.74	
	(AG + CC)	183.42 ± 41.27	
	(AG + AA)	166.94 ± 47.61	
	(GG + AA)	176.92 ± 45.11	

Triglycerides	(AA + AA)	277.84 ± 133.61	0.45
	(AG + AC)	317.14 ± 125.36	
	(GG + CC)	274.09 ± 109.46	
	(AG + CC)	336.88 ± 123.27	
	(AG + AA)	289.20 ± 128.33	
	(GG + AA)	313.30 ± 120.98	

Systolic BP	(AA + AA)	132.08 ± 24.79	0.28
	(AG + AC)	138.63 ± 21.56	
	(GG + CC)	133.36 ± 25.96	
	(AG + CC)	141.96 ± 22.52	
	(AG + AA)	134.87 ± 24.31	
	(GG + AA)	138.53 ± 23.17	

Diastolic BP	(AA + AA)	93.84 ± 22.38	0.010^*∗*^
	(AG + AC)	100.10 ± 17.54	
	(GG + CC)	95.45 ± 21.38	
	(AG + CC)	100.39 ± 16.99	
	(AG + AA)	93.22 ± 19.21	
	(GG + AA)	94.87 ± 15.87	

^*∗*^Significant at *p* > 0.05.

**Table 8:** Regression analysis: dependent variable cases and control.

Variables	*R* ^2^	*p* value
Age	0.05	<0.0001
Gender	0.006	0.11
BMI	0.119	<0.0001
PPG	0.360	<0.0001
FPG	0.317	<0.0001
FPI	0.099	<0.0001
HbA1C	0.290	<0.0001
Cholesterol	0.862	<0.0001
HDL	0.00	0.80
LDL	0.689	<0.0001
Systolic BP	0.560	<0.0001
Diastolic BP	0.378	<0.0001
Triglycerides	0.535	<0.0001
Cases and controls as dependent variables		

^*∗*^Significant at *p* > 0.05.

## Data Availability

We confirm that the data used during the research will not be shared with anybody or broadcasted in any public domain, since it is impermissible as per the policy instructions of JMI. The metadata, supporting the study outcomes, can be accessed from JMI through proper consent; however, privileged data with restricted open accessibility, cedes institutional authorization, and discretion and other related information and data can also be retrieved from the authors with permission of JMI.

## Abbreviations

IDF: International Diabetes Federation
KCNJ11: Potassium inwardly rectifying channel, subfamily J, member 11
KCNQ1: Potassium voltage-gated channel KQT-like subfamily, member 1
GWAS: Genome-wide association studies
PCR-RFLP: Polymerase chain reaction-restriction fragment length polymorphism
SUR1: Sulfonylurea receptor 1
KATP: ATP- sensitive K channel
OR: Odds ratio
CI: Confidence interval
HWE: Hardy–Weinberg equilibrium
SNP: Single nucleotide polymorphism.

## Additional Points

*Highlights*. (i) The genotype and allele frequencies in type 2 diabetes cases were significantly different compared to the control population. (ii) Clinicopathological parameters were analysed, and a significant association was found between the carrier of a variant allele with gender and PPG in KCNJ11 and with triglyceride in KCNQ1. (iii) KCNJ11 (rs5210) and KCNQ1 (rs2237895) gene variants are significantly associated with type 2 diabetes in the Indian population.
